# CARK3-mediated ADF4 regulates hypocotyl elongation and soil drought stress in Arabidopsis

**DOI:** 10.3389/fpls.2022.1065677

**Published:** 2022-12-21

**Authors:** Lu Peng, Juan He, Huan Yao, Qin Yu, Qian Zhang, Kexuan Li, Yaling Huang, Li Chen, Xufeng Li, Yi Yang, Xiaoyi Li

**Affiliations:** Key Laboratory of Bio-Resources and Eco-Environment of Ministry of Education, State Key Laboratory of Hydraulics and Mountain River Engineering, College of Life Sciences, Sichuan University, Chengdu, China

**Keywords:** hypocotyl elongation, drought tolerance, actin depolymerization factor, CARK3, phosphorylation

## Abstract

Actin depolymerization factors (ADFs), as actin-binding proteins, act a crucial role in plant development and growth, as well as in response to abiotic and biotic stresses. Here, we found that CARK3 plays a role in regulating hypocotyl development and links a cross-talk between actin filament and drought stress through interaction with ADF4. By using bimolecular fluorescence complementation (BiFC) and GST pull-down, we confirmed that CARK3 interacts with ADF4 *in vivo* and *in vitro*. Next, we generated and characterized double mutant *adf4cark3-4* and OE-ADF4:*cark3-4*. The hypocotyl elongation assay indicated that the *cark3-4* mutant seedlings were slightly longer hypocotyls when compared with the wild type plants (WT), while *CARK3* overexpressing seedlings had no difference with WT. In addition, overexpression of *ADF4* significantly inhibited long hypocotyls of cark3-4 mutants. Surprisingly, we found that overexpression of *ADF4* markedly enhance drought resistance in soil when compared with WT. On the other hand, drought tolerance analysis showed that overexpression of *CARK3* could rescue *adf4* drought susceptibility. Taken together, our results suggest that CARK3 acts as a regulator in hypocotyl elongation and drought tolerance likely *via* regulating ADF4 phosphorylation.

## Introduction

Actin filaments (F-actin), as a scaffold in cells, play a fundamental role in numerous cellular processes, including morphogenesis, motility and cell elongation. In addition, the studies have shown that F-actin also functions in response to abiotic and biotic stresses in planta (Lin et al., 2014). The dynamic of F-actin is regulated by actin-binding proteins (ABPs), including actin depolymerization factors (ADFs), formin family proteins and adenylyl cyclase-associated proteins (Liu et al., 2018; Jiang et al., 2019).

ABPs are involved in regulating a vast array of fundamental biological processes, such as the normal organism development (Ketelaar et al., 2004). For example, the actin-binding protein Rice Morphology Determinant (RMD) in rice (*Oryza sativa*) promotes statolith mobility in gravisensing endodermal cells, and for proper auxin distribution in light-grown shoots through reorganization of the actin cytoskeleton (Song et al., 2019). ADF7 and ADF10 directly modulate pollen tube elongation in Arabidopsis (Zheng et al., 2013; Jiang et al., 2017; Jiang et al., 2022). UV-B inhibits Arabidopsis hypocotyl elongation due to the fact that actin filaments shift from bundles to a loose arrangement (Du et al., 2020b). PRF4 and PRF5, as actin-binding proteins, could regulate vesicle trafficking and polarity establishment during pollen germination by enhancing the interaction between AtFH5 (Arabidopsis formin homology 5) and actin filaments (Liu et al., 2021). In the dark, *ADF4* mutants have longer hypocotyl and epidermal cells when compared with wild-type seedlings (Henty et al., 2011; Yao et al., 2022). However, the molecular mechanism of ADF4 is not uncovered in regulating the hypocotyl elongation.

In addition, ABPs have been implicated in integration of cellular responses to extracellular and intracellular signals during biotic and abiotic stresses. *GhVLN4* from cotton, as one type of major ABPs responsible for microfilament bundling, overexpression of which shows resistance to *Verticilium dahliae*, salt and drought stresses in Arabidopsis. (Ge et al., 2021). *Actin-depolymerizing factor 1* (ADF) is repressed by MYB73, which is known as a negative regulator in salt tolerance, and then regulates actin filaments organization (Wang et al., 2021). Additionally, ADF4 is mediated by CPK3 to control pathogen-induced actin reorganization during immune signaling, and phosphorylated by CKL2 to regulate actin filaments in stomata in response to dehydration (Guo et al., 2016; Lu et al., 2020). However, the recent studies have revealed that ADF5 has evolved F-actin-bundling activity, thus positively regulates drought stress (Qian et al., 2019). *DaADF3* from *Deschampsia antarctica* is induced by drought stress (Byun et al., 2021). Knockdown of *TaADF3* from wheat leads to *Puccinia striiformis* susceptibility, which is accompanied by increased ROS production (Tang et al., 2015). ADFs regulate the dynamics of actin filaments in response to environmental cues.

Our previous studies have shown that CAK3 kinase protein could form dimer to phosphorylate ABA (abscisic acid) receptors, and subsequently trigger ABA signaling pathway (Li et al., 2022). Here, we observed that *CARK3* T-DNA mutants exhibited long hypocotyl compared with the wild-type plants. Further, we found that CARK3 phosphorylates ADF4, and the association cooperatively mediates the hypocotyl growth in the dark. Moreover, the interaction between CARK3 and ADF4 led us to investigate a role of CARK3 during drought stress. The evaluation of drought tolerance reveals that overexpression of *ADF4* could resume *cark3-4* drought sensitivity in Arabidopsis.

## Materials and methods

### Plant materials and growth conditions

Plant materials and growth conditions Arabidopsis plants used in this study were all in the Columbia (Col-0) background. The *cark3-4* and *adf4* T-DNA insertion mutants, OE-*ADF4* #10, #17, #18 and *CARK3*-OE 2 overexpression plants were previously described (Wang et al., 2020; Yao et al., 2022). Plants were grown on soil-vermiculite mixtures at 22°C under 60% relative humidity with cycles of 16 h light and 8 h dark. For plate experiment, seeds were stratified in distilled water for 3 days at 4°C in dark. The imbibed seeds were surface sterilized with a 20% bleach solution for 15 min and then washed 5 times with sterilized water, and then sown on Murashige and Skoog (MS) medium containing 2% sucrose and 1.0% agar, pH 5.8.

### Bimolecular fluorescence complementation (BiFC) assay

The ADF4-YFP^N^ and CARK3-YFP^C^ constructs were previously described (Wang et al., 2020; Yao et al., 2022). The constructs were transformed into Agrobacteria GV3101 stain. And then the indicated YFP^N^/YFP^C^ combination were co-expressed in 4-week old tobacco leaves. After 2 days, the signals of YFP were observed with a Leica confocal laser scanning microscope (DM4 B). 3 leaves were observed each time and three biological repeats were performed.

### GST pull-down assay

To test the interaction of CARK3 with ADF4 *in vitro*, GST-CARK3, GST and His-ADF4 were purified as previously described (Li et al., 2022; Yao et al., 2022). 5 μg GST-CARK3 or GST bound glutathione-agarose beads were incubated with His-ADF4 in binding buffer (50 mM Tris-HCl, pH 8.0, 200 mM NaCl, 10% glycerol, 0.1% Tween 20) at room temperature for 30 min and washed 10 times with binding buffer. The pulled-down compounds were eluted with 2× SDS sample buffer (24 mM Tris-Cl, pH 6.8, 10% glycerol, 0.8% SDS, and 2% 2-mercaptoethanol) and boiled at 98°C for 10 min. Next, proteins were analyzed by immunoblotting with GST and His antibodies.

### *In vitro* kinase assay

To investigate CARK3-mediated phosphorylation of ADF4, the purified ADF4 was incubated with His-CARK3-KD (Kinase domain) in kinase buffer (20 mM Tris, pH 7.5, 1 mM MgCl_2_, 100 mM NaCl, and 1 mM DTT, 5 mM ATP), which was previously described (Wang et al., 2020). After incubation at 30°C for 30 min, the reaction mixture was terminated by adding an equal volume of 2 × SDS-PAGE (Sodium Dodecyl Sulfate, Polyacrylamide Gel Electrophoresis) loading buffer, and boiled at 98°C for 5 min. The proteins were separated by SDS–PAGE and analyzed with Anti-Phosphoserine/threonine Rabbit Polyclonal (Cell Signaling Technology).

### Physiological analysis

To test the interaction of CARK3 with ADF4 in response to soil drought stress, we obtained the double mutant *adf4cark3-4* and OE-*ADF4*:*cark3-4* by crosses. For drought tolerance experiment, 7 d seedlings indicated genotypes were grown under the same conditions and then subjected to drought stress treatment by withholding water for 12 or 13 days. Then, 2 days after rehydration, the morphological changes of plants were recorded, including survival rate and fresh weight (FW).

For hypocotyl elongation assay, genotypes were grown in the dark for 4 d after germination and then representative seedlings were photographed. At least 20 seedlings per genotype were measured as one biological replicate using ImageJ software (http://imagej.nih.gov/ij/).

Stomatal aperture was carried out as previously described (Li et al., 2022). 3-week old leaves were peeled and then incubated in opening buffer (50 mM KCl, 10 μM CaCl_2_, 10 mM MES pH 6.15) for 3 h to induce stomatal opening. For ABA treatment, 5 and 20 μM ABA was added to the opening buffer for 30 min or 2 h. Images were taken using Leica TCS SP5 II HCS confocal microscope.

For nitroblue tetrazolium (NBT) staining to test superoxides, 3-week-old leaves were treated with or without 50 μM ABA for 1 days, and then incubated in NBT buffer for overnight. Chlorophyll was removed with 95% ethanol.

### Statistical analysis

In this study, statistical data were analyzed using GraphPad Prism version 8 software (GraphPad Software, La Jolla, CA, USA). Data were collected in triplicates and subjected to analysis of variance (ANOVA). *Post hoc* means separation was done with Tukey’s test at P < 0.05.

## Results

### CARK3 is involved in hypocotyl elongation

**Figure 1:**
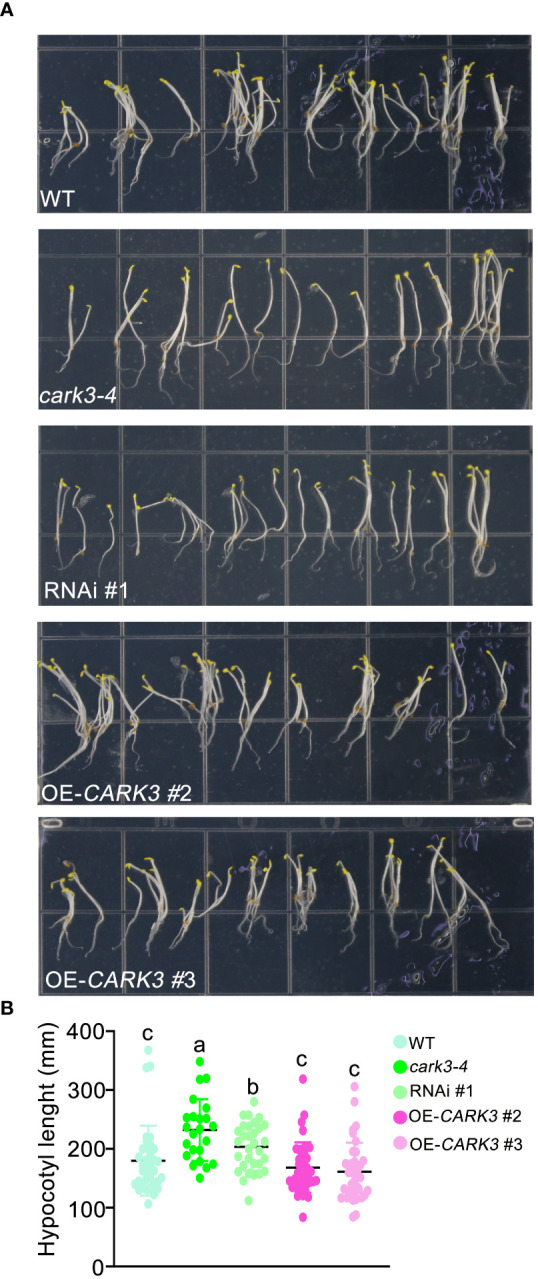
CARK3 is involved in hypocotyl elongation. **(A)** Seedlings from wild-type plants, CARK3 T-DNA insertion mutant (*cark3-4*), RNAi #1 plant and *CARK3*-overexpressing lines (*CARK3*-OE 2/3) grown on 1/2 MS growth medium in the dark for 4 d. **(B)** The graph shows the average hypocotyl length measured from a minimum of 20 seedlings. Each point represents an individual seedling. Lines and error bars represent the mean and 95% confidence interval for each genotype, respectively. Bars with different alphabets indicate significant difference by Tukey test at P < 0.05, while those with a common alphabet indicate no significant difference (P > 0.05).

In this study, we found that *cark3-4* T-DNA mutants and RNAi plants exhibited longer length of hypocotyl than WT (**Figure 1**). In addition, the overexpression of *CARK3* lines had markedly shorter hypocotyl when compared with the wild-type plants (**Figure 1**). These results suggest that CARK3 modulates hypocotyl growth in Arabidopsis.

### CARK3 phosphorylates ADF4

**Figure 2:**
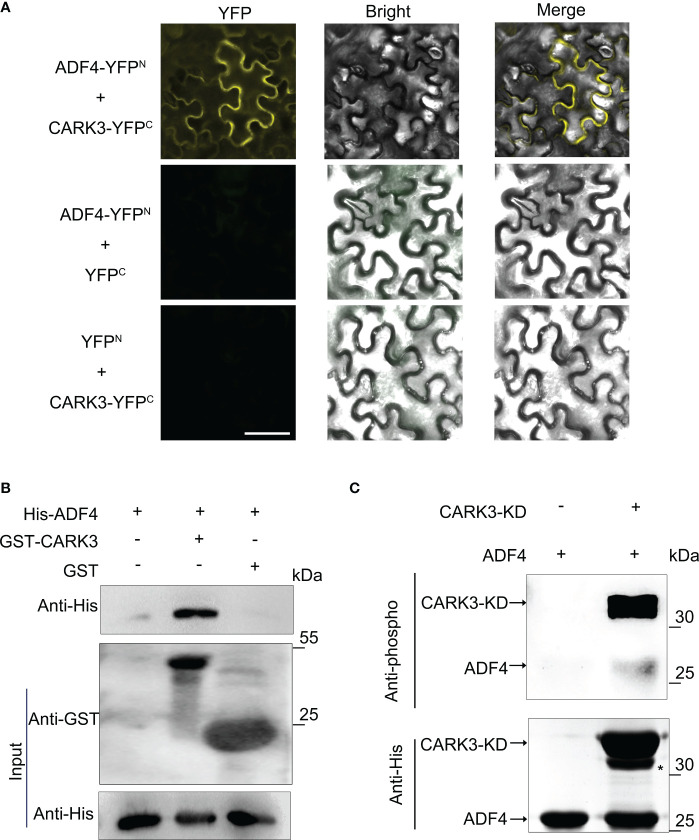
CARK3 interacts with ADF4. **(A)** BiFC assays showing interaction between CARK3 and ADF4 in tobacco leaves. Negative controls used were ADF4-YFP^N^ +YFP^C^ and YFP^N^ +CARK3-YFP^C^. Scale bars = 50 μm. **(B)** Pull-down assays showing that GST-CARK3 interacts with His-ADF4. His-ADF4 pulled down by GST-CARK3 was detected by anti-His antibody. GST was used as a negative control and did not interact with His-ADF4. **(C)** *In vitro* phosphorylation of ADF4 by CARK3. Western blot assay with anti-phosphoserine/threonine antibody. Anti-His antibody was used to test loading.

In our previous study, we reported that 14-3-3κ coordinates ADF4 to regulate hypocotyl elongation (Yao et al., 2022). The 14-3-3 proteins usually interact with kinase proteins to mediate signal transduction by altering their activity, or affinity to other proteins (Paul et al., 2012). Thus, we proposed that CARK3 would interact with 14-3-3κ. However, the bimolecular fluorescence complementation (BiFC) assay showed that CARK3 could not interact with 14-3-3κ *in vivo*. Next, we tested the association of CARK3 with ADF4 using the BiFC assay. The yellow fluorescent signals were observed in tobacco cells when co-expression of ADF4-YFP^N^ and CARK3-YFP^C^. However, no signal was found, when co-expression of ADF4-YFP^N^ and YFP^C^ or YFP^N^ and CARK3-YFP^C^ (**Figure 2A**). These results indicate that CARK3 interact with ADF4 *in vivo*.

To further confirm the physical interaction of CARK3 and ADF4, we then performed an *in vitro* pull-down assay using bacterially expressed purified proteins. GST and His-ADF4, GST-CARK3 and His-ADF4 were co-incubated with GST resin, respectively. The bound proteins were tested with anti-GST and anti-His antibodies, respectively. As indicated in **Figure 2B**, GST-CARK3 could pull-downed His-ADF4, while GST could not, indicating that CARK3 physically interacts with ADF4 *in vitro*.

CARK3 has been shown to phosphorylate ABA receptors to mediate ABA signaling pathway (Li et al., 2022). ADF4 has revealed to be phosphorylated by CKL2 (*Arabidopsis thaliana* casein kinase 1-like protein 2) and CPK3 (Arabidopsis calcium-dependent protein kinase 3), showing multiple potential Ser/Thr phosphorylation sites (Guo et al., 2016; Lu et al., 2020). Thus, ADF4 could be a substrate of CARK3. To address this, an *in vitro* kinase assay was conducted. The phosphorylation band was observed, when ADF4 was incubated with CARK3. However, no band was detected in the gel without CARK3 (**Figure 2C**). The results suggest that CARK3 phosphorylates ADF4 *in vitro*.

### CARK3-mediated ADF4 regulates hypocotyl elongation

**Figure 3:**
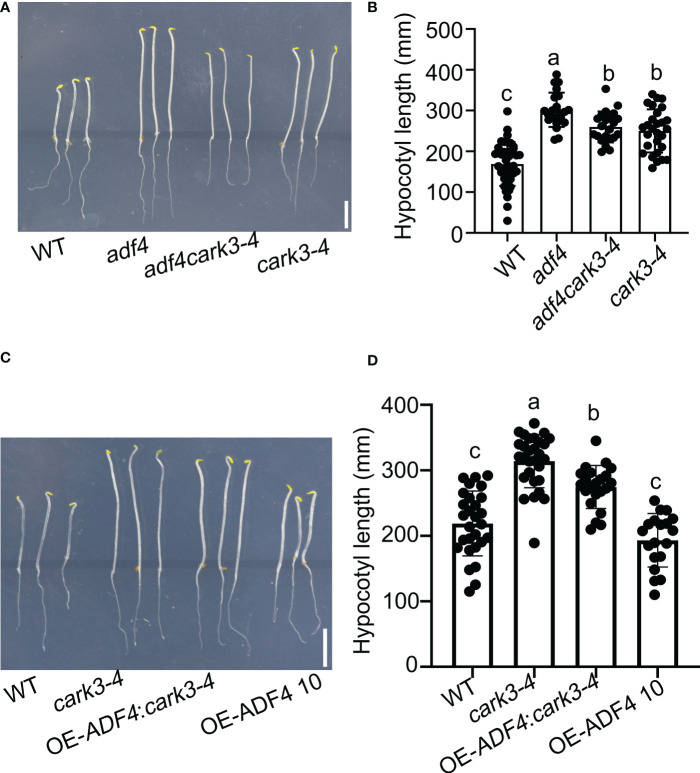
Overexpression of *ADF4* partially inhibited hypocotyl growth of c*ark3-4* mutants. **(A)** *adf4cark3-4* seedlings developed shorter hypocotyls when compared with *adf4* mutants. **(C)** OE-*ADF4*: *cark3-4* seedlings developed shorter hypocotyls than *cark3-4* mutants. **(B, D)** The graphs shows the average hypocotyl length measured from a minimum of 20 seedlings in **(A)** and **(C)**, respectively. Each point represents an individual seedling. Lines and error bars represent the mean and 95% confidence interval for each genotype, respectively. Bars with different alphabets indicate significant difference by Tukey test at P < 0.05, while those with a common alphabet indicate no significant difference (P > 0.05).

To investigate ADF4 phosphorylated by CARK3 in regulation of hypocotyl elongation, we generated *adf4cark3-4* double mutant and OE-*ADF4*:*cark3-4* by crossing *cark3-4* with *adf4* and OE-*ADF4*, respectively (Yao et al., 2022). Next, sterilized seeds from WT, *adf4*, *cark3-4*, *adf4cark3-4* and OE-*ADF4*:*cark3-4* were plated on 1/2 MS plates grown vertically in the dark. After 4 d, we observed that *adf4cark3-4* double mutants displayed longer hypocotyls when compared with the *cark3-4* plants, and no significant difference with *adf4* or *cark3-4* (**Figures 3A, B**). On the other hand, the hypocotyl length in the *cark3-4* mutant was shorter than that of the OE-*ADF4*:*cark3-4* seedlings, which had longer hypocotyl length than OE-*ADF4* transgenic plants (**Figures 3C, D**). The hypocotyl length of the *adf4* seedlings exhibited longer than that of WT, which is consistent with the previous report (Henty et al., 2011; Yao et al., 2022). Considering that CARKs are involved in ABA signaling, we also investigated ABA-mediated hypocotyl growth in darkness. The results showed that *cark3-4* seedlings were insensitive to ABA (**Figure S1**), which may due to the fact that *cark3-4* plants have higher germination late that WT under ABA treatment (Wang et al., 2020). These results suggest that the phosphorylation of ADF4 by CARK3 mediates hypocotyl elongation in Arabidopsis.

### Overexpression of *ADF4* enhances long-term drought tolerance

**Figure 4:**
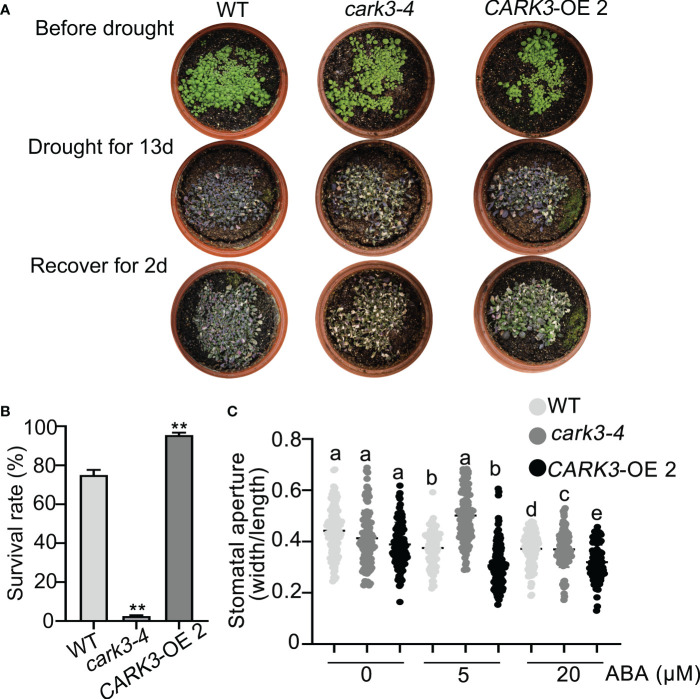
Overexpression of *CARK3* enhance Arabidopsis drought tolerance. **(A)** The representative images of the plants from WT, *cark3-4* and *CARK3*-OE that were grown for 7 days under normal conditions, then treated for 13 days with drought, and rewatered for 2 days. **(B)** Quantitative analysis of survival rates in **(A)**. Mean ± SEM, n = 3. ^**^ *P* < 0.05, Student’s *t*-test. The experiment was repeated three times, at least five pots at a time. **(C)** ABA-induced stomatal closure was measured with or without ABA treatment for 30 min. Low (5 μM) or high (20 μM) concentrations of ABA were added to the opening buffer. Each point represents some individual stomata, at least 50 stomata per genotype were measured. Bars with different alphabets indicate significant difference by Tukey test at P < 0.05, while those with a common alphabet indicate no significant difference (P > 0.05).

**Figure 5:**
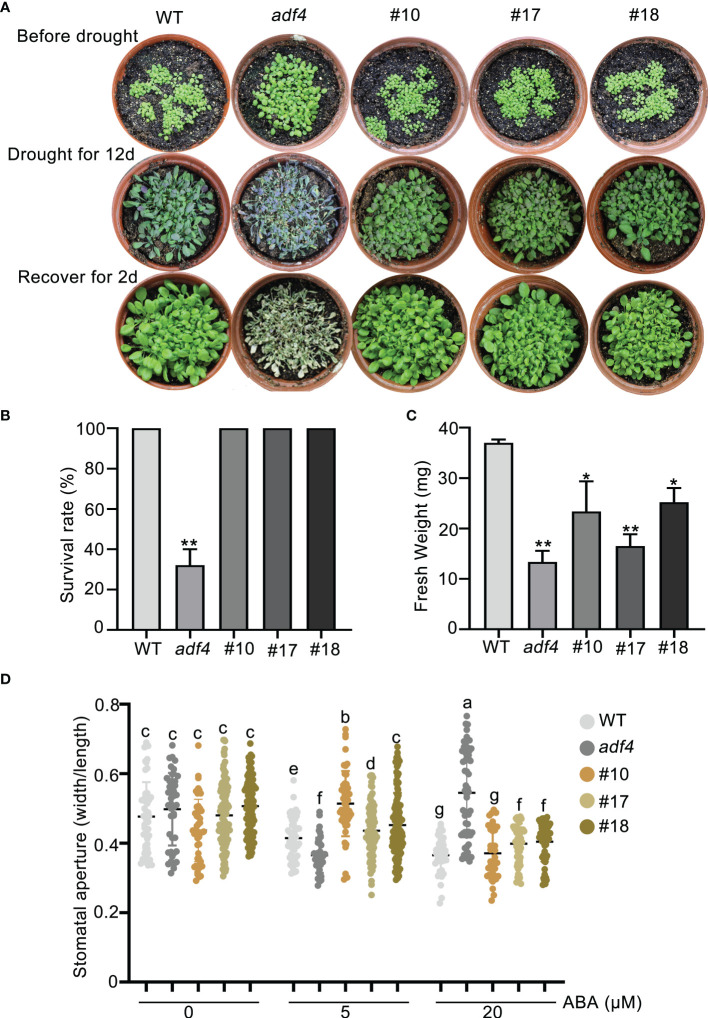
Overexpression of *ADF4* enhance Arabidopsis drought tolerance. **(A)** Images of plants before and after drought and after rewatering, including WT, *adf4* and OE-*ADF4* lines. The plants were grown for 7 days under normal conditions, then treated for 12 days without watering and rewatered for 2 days. **(B, C)** Quantities of survival rates and fresh weight of per pot from genotypes after rewatering in **(A)**. The error bars indicate the SEM from three replicates (n = 3). ^*^ *P* < 0.05, ^**^ *P* < 0.01, Student’s *t*-test. The experiment was repeated three times, at least three pots at a time. **(D)** Stomatal aperture of WT, *adf4* and overexpression of *ADF4* plants. Four-week-old Arabidopsis leaves were treated with or without low (5 μM) and high (20 μM) concentrations of ABA for 2 h, respectively. Each point represents some individual stomata, at least 50 stomata per genotype were measured (the ratio of width to length). Bars with different alphabets indicate significant difference by Tukey test at P < 0.05, while those with a common alphabet indicate no significant difference (P > 0.05). #10, #17 and #18 represent the overexpression of *ADF4* lines in wild-type plants.

The previous studies have shown that CARK3 phosphorylates ABA receptors, triggers ABA binding, resulting in plant resistance to drought stress (Wang et al., 2020). In addition, ADF4 is also involved in stomatal closure through reorganization actin filaments (Guo et al., 2016). Thus, we proposed that CARK3 participates in drought tolerance, through regulation of ADF4 activity. To test this, we performed the drought tolerance assay in soil. The results showed that *CARK3*-OE plants had higher survival rate, while *cark3-4* mutants had lower when compared with the wild-type (WT) plants (**Figures 4A, B**). We further examined stomatal closure in response to ABA. The data showed that the *cark3-4* mutant was insensitive to ABA-induced stomatal closure under low (5 μM) and high (20 μM) concentration of ABA treatment (**Figure 4C**). Interestingly, we found that OE-*ADF4* lines were similar to WT, with 100% survival rate (**Figures 5A, B**), while the percent survival of *adf4* was significantly lower than those of WT after drought treatment. The results of fresh weight also confirmed the phenotype analysis (**Figure 5C**). Additionally, the *adf4* mutant was sensitive to the low concentration of exogenous ABA (**Figure 5D**), which is consistent with the previous report (Guo et al., 2016). However, we here found that the *adf4* mutant was insensitive to ABA-induced stomatal closure, the plants overexpressing *ADF4* were sensitive under the high concentration of exogenous ABA treatment (**Figure 5D**). These data reveal that CARK3 and ADF4 are positive regulators in response to soil drought stress in Arabidopsis.

### Drought resistance by CARK3 is dependent on ADF4

**Figure 6:**
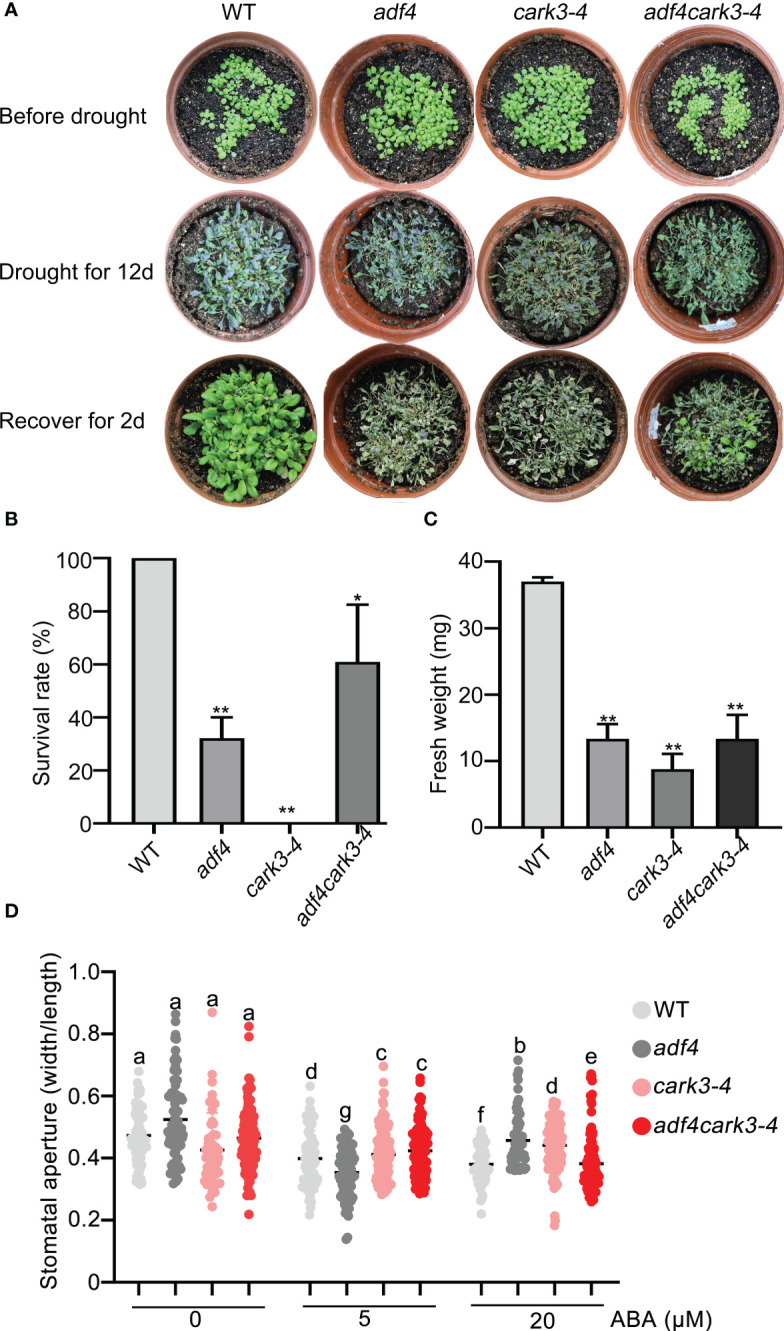
*adf4* partially resumes *cark3* in response to soil drought stress. **(A)** Phenotypic comparison of WT, *cark3-4*, *adf4* and *adf4cark3-4* plants (upper) grown in soil after water was withheld for 12 days (middle) and the plants were then rewatered for 2 day (bottom). Three independent experiments were performed that yielded similar results; 5 pots per genotype for each time. **(B, C)** The survival rates and fresh weight were assessed in three replicates (n = 3). ^*^ *P* < 0.05, ^**^ *P* < 0.05, Student’s *t*-test. **(D)** Stomatal closure (the ratio of stomatal width/length) was measured in WT, *cark3-4*, *adf4* and *adf4cark3-4* plants in response to 5 μM and 20 μM exogenous ABA. Each point represents some individual stomata, at least 50 stomata per genotype were measured. Bars with different alphabets indicate significant difference by Tukey test at P < 0.05, while those with a common alphabet indicate no significant difference (P > 0.05).

**Figure 7:**
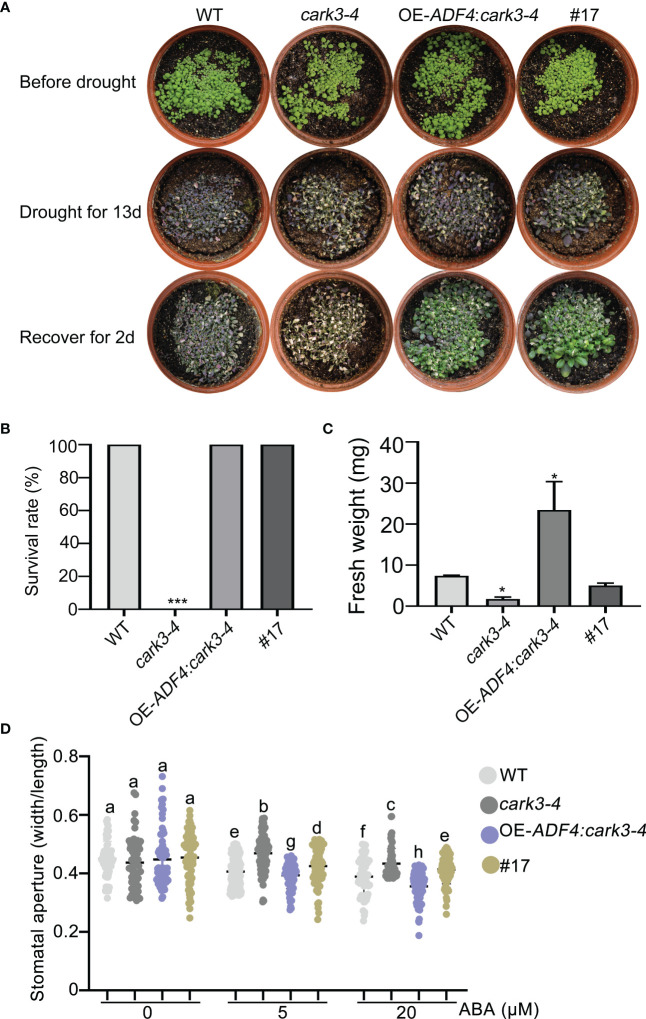
Overexpression of *CARK3* rescues *adf4* drought sensitivity. **(A)** Images of plants before and after drought and after rewatering, including WT, *cark3-4* and OE-*ADF4*: *cark3-4* genotypes. The plants were grown for 7 days under normal conditions, then treated for 13 days with drought, and rewatered for 2 days. **(B, C)** The survival rates and fresh weight were assessed in three replicates (n = 3). ^*^ *P* < 0.05, ^***^ *P* < 0.001, Student’s *t*-test. **(D)** ABA-induced stomatal closure was measured in each plant indicated under low (5 μM) and high (20 μM) concentrations of ABA treatment for 2 h. Each point represents some individual stomata, at least 50 stomata per genotype were measured. Bars with different alphabets indicate significant difference by Tukey test at P < 0.05, while those with a common alphabet indicate no significant difference (P > 0.05).

To determine the genetic relation of CARK3 with ADF4 in drought resistance, we performed drought tolerance experiment. The results showed that the *adf4cark3-4* double mutant had lower level of survival rate than WT, which had 100% survival rate and higher level than that of *adf4* or *cark3-4* genotype (**Figures 6A, B**). Similarly, WT plants had highest fresh weight after rewatered following 12-day dehydration (**Figure 6C**). ABA-induced stomatal closure assay also confirmed that the loss of *ADF4* rescued the ABA insensitivity of *cark3-4* mutant under both low and high levels of exogenous ABA treatment (**Figure 6D**). Next, we analyzed OE-*ADF4*:*cark3-4* plants in drought tolerance compared with OE-*ADF4*, *cark3-4* and WT, respectively. The results showed that the percent survival rate of WT, OE-*ADF4* and OE-*ADF4*:*cark3-4* plants were indistinguishable (**Figure 7A**). However, OE-*ADF4*:*cark3-4* showed better and had higher level of fresh weight when compared WT OE-*ADF4* plants (**Figures 7B, C**). For ABA-induced stomatal aperture assay, we observed that the stomatal sensitivity of OE-*ADF4*:*cark3-4* plants to ABA recovered to that of the WT plants after low concentration of ABA treatment (**Figure 7D**). ABA induced-stomatal closure under drought stress also contributes to the production of hydrogen peroxide (H_2_O_2_) (An et al., 2019; Pei et al., 2022). In this study, we found that H_2_O_2_ accumulation in *adf4* mutants was higher than that in WT, while the *ADF4*-overexpressing line (#17) produced less H_2_O_2_ after ABA treatment (50 μM) (**Figure S2**). These phenotypic data indicate overexpression of *ADF4* mediates long-term drought tolerance through the its phosphorylation by CARK3.

## Discussion

Plants need to respond to photomorphogenesis when grown in the light, and also undergo skotomorphogenesis in the dark, exhibiting long hypocotyls, as well as survive in response to multiple developmental and environmental cues. Hypocotyl length is usually controlled by cell elongation which is regulated by the dynamics of actin filaments (Fan et al., 2013; Zhao et al., 2015; Du et al., 2020b). In this study, we demonstrated that the phosphorylation of ADF4 mediated by CARK3 controls hypocotyl cell elongation and modulates soil dehydration in Arabidopsis.

Previous studies have revealed cross-talk between auxin and ABA signaling transduction (Emenecker et al., 2021). Auxin could induce reorganization of actin filaments (Du et al., 2020a). Loss of *AUX1* (auxin transporter AUXIN RESISTANT 1) leads to failure of actin reorganization in response to IAA treatment (Arieti and Staiger, 2020). ADF4 modulates auxin distribution and transport through regulating the dynamics of actin filaments, which is required for cell expansion (Deeks and Hussey, 2009; Jiang et al., 2020). Additionally, ABA receptors PYL8 (RCAR3) and PYL9 (RCAR1) could interact with the transcription factor PIF3 to regulate hypocotyl elongation (Qi et al., 2020). Our previous study shows that CARKs phosphorylate ABA receptors (Zhang et al., 2018). Thus, the interaction of ADF4 and CARK3 offers exciting new insights into the cross-talk between ABA signaling and actin filament in hypocotyl elongation.

The previous studies have revealed that *adf4* mutants have smaller stomatal aperture than the wild-type plants under 2 μM ABA treatment, indicating that ADF4 acts a negative regulator in ABA-mediated drought tolerance (Guo et al., 2016). However, *adf4* mutants displayed lower survival rate compared with WT, when genotypes were subjected to long-term drought stress (**Figure 5**). We inferred that the inhibition of ADF4 activity by CARK3, leading to fail actin filament disassembly during stomatal closure. However, CKL2 inhibits ADF4 activity on severing actin filaments, leading to actin arrays being reorganized into highly bundled long cables and maintain stomatal closure (Guo et al., 2016). The clade A PP2C phosphatase would inhibit CKL2 kinase activity, when water is supplied (Shi et al., 2021). Thus, overexpression of *ADF4* triggers F-actin in guard cell from cortical filaments to randomly distributed (Hwang and Lee, 2001), then CKL2 and CARK3 inhibit ADF4 activity, and then actin filaments changes to longitudinal direction, which stabilizes guard cells in closed stomata. Similarly, CPK3-mediated ADF4 governs actin filaments organization and activate pattern- and effector-triggered immunity (Lu et al., 2020). In summary, ADF4 is mediated by upstream regulators during plant development and in response to abiotic and biotic stresses.

## Data availability statement

The original contributions presented in the study are included in the article/**Supplementary Material**. Further inquiries can be directed to the corresponding author.

## Author contributions

XYL and YY conceived and designed the experiment. LP and JH conducted the BiFC analysis. HY and QY preformed Arabidopsis crossing. KL, YH, and LC conducted drought tolerance experiments. XFL, YY, and XYL analyzed the results and wrote the paper. All authors contributed to the article and approved the submitted version.

## Supplementary material

Supplementary Figure 1 Statistical analysis of hypocotyl length of seedlings in darkness. Seeds were sown on plants containing 0.1 μM ABA. Over 20 seedlings per genotype were measured after 4 d. Violin plot showing the average stomatal aperture (width/length). Student’s *t*-test, ^*^ *P* < 0.05.

Supplementary Figure 2 ABA-induced ROS accumulation. NBT staining indicates different levels of ABA-induced H_2_O_2_ production in leaves of the plants indicated. To detect O_2_^-^, 4-week-old plant leaves were staining with NBT after 2 days with or without 50 μM ABA.
