# Supplementary figures and images for: CARK3-mediated ADF4 regulates hypocotyl elongation and soil drought stress in Arabidopsis
Source: Front Plant Sci. 2022 Dec 21;13:1065677. doi: 10.3389/fpls.2022.1065677 (PMC9811263; doi:10.3389/fpls.2022.1065677)

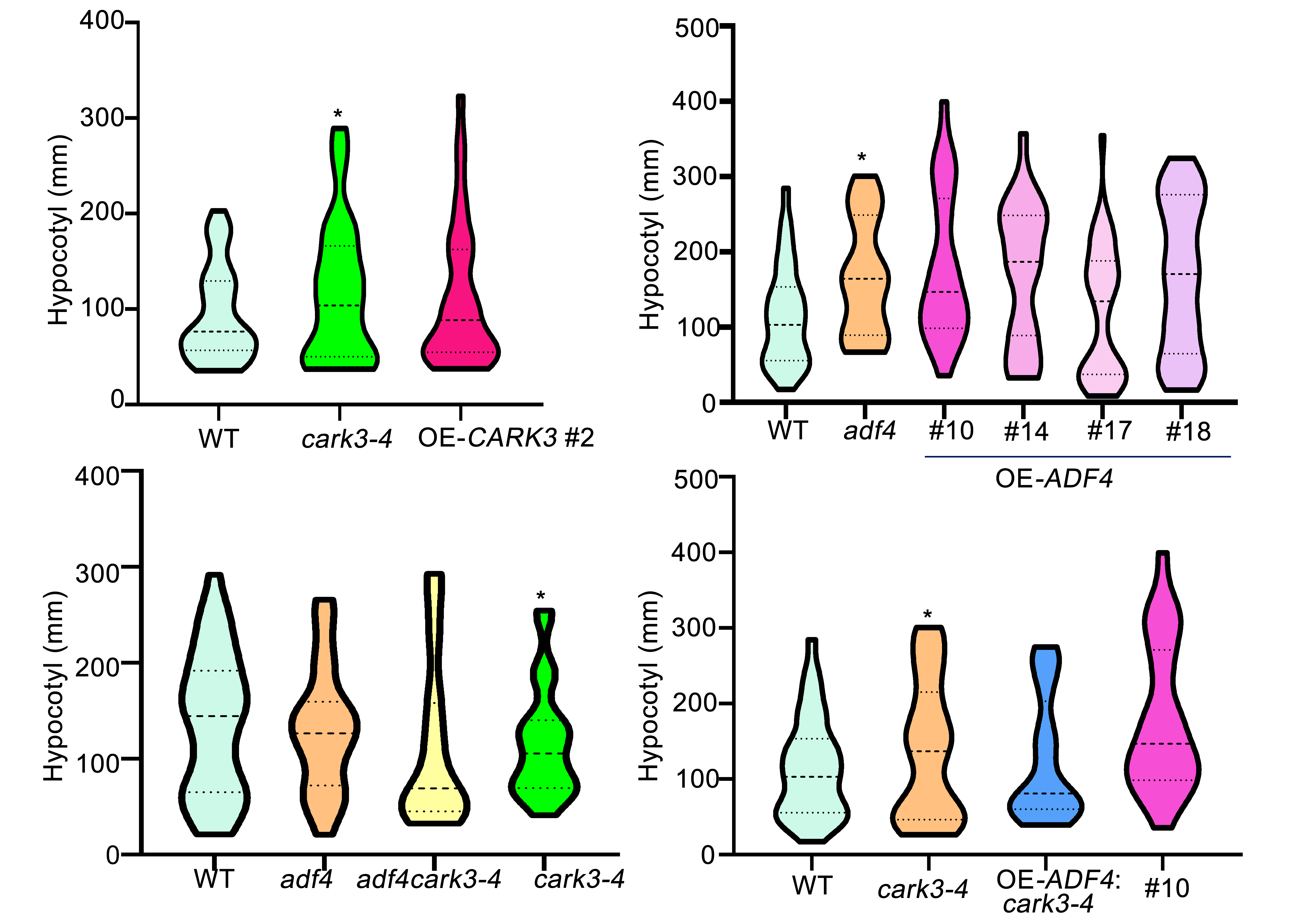

Supplement: Supplementary Figure 1 — Statistical analysis of hypocotyl length of seedlings in darkness. Seeds were sown on plants containing 0.1 μM ABA. Over 20 seedlings per genotype were measured after 4 d. Violin plot showing the average stomatal aperture (width/length). Student’s t-test, * P < 0.05. [file Image_1.tif]

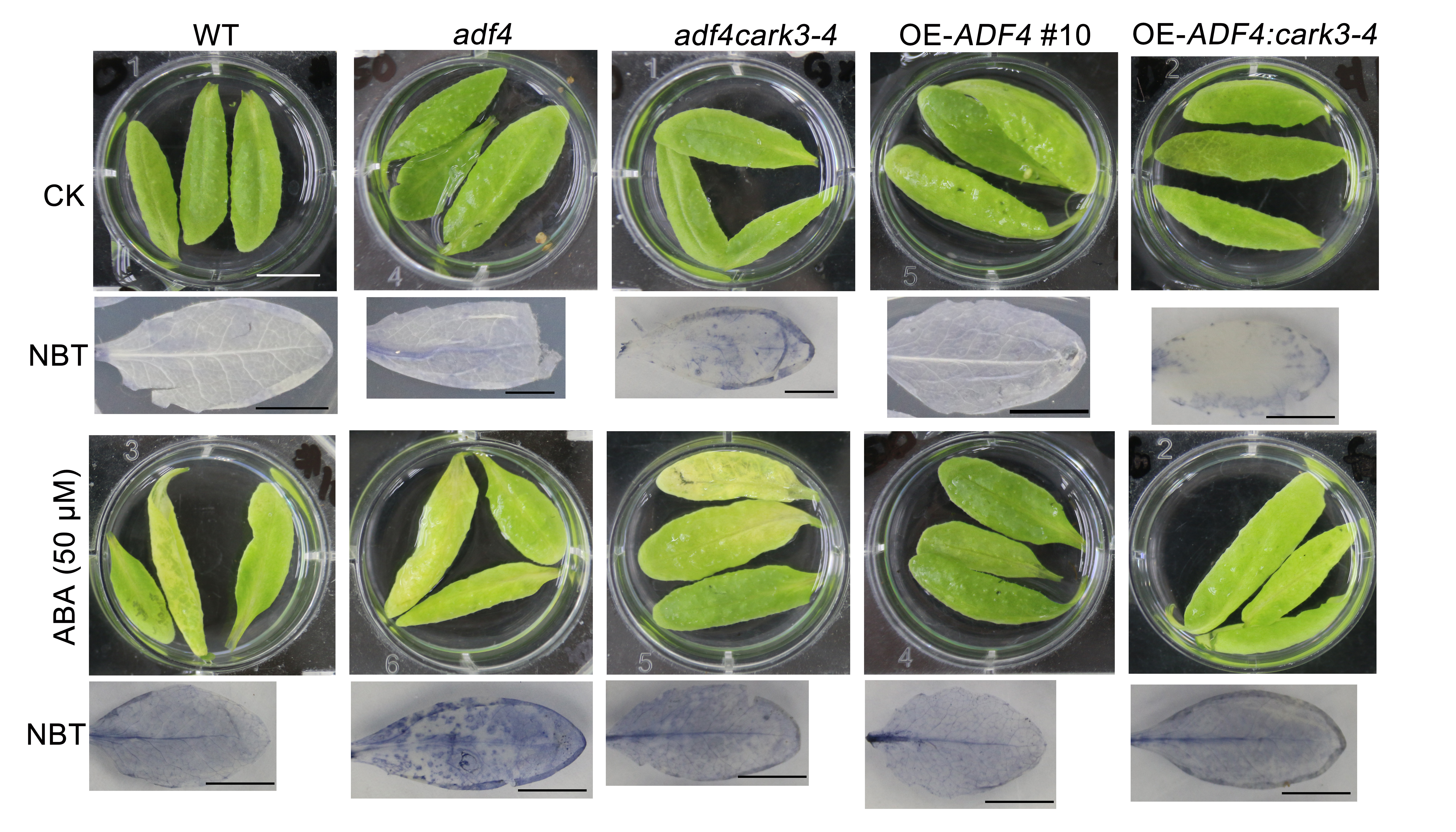

Supplement: Supplementary Figure 2 — ABA-induced ROS accumulation. NBT staining indicates different levels of ABA-induced H2O2 production in leaves of the plants indicated. To detect O2 -, 4-week-old plant leaves were staining with NBT after 2 days with or without 50 μM ABA. [file Image_2.jpeg]
